# Prediction and classification of Alzheimer disease based on quantification of MRI deformation

**DOI:** 10.1371/journal.pone.0173372

**Published:** 2017-03-06

**Authors:** Xiaojing Long, Lifang Chen, Chunxiang Jiang, Lijuan Zhang, Alzheimer’s Disease Neuroimaging Initiative

**Affiliations:** 1 Paul C. Lauterbur Research Center for Biomedical Imaging, Shenzhen Institutes of Advanced Technology, Chinese Academy of Sciences, Shenzhen, Guangdong, China; 2 Department of Neurology, Shenzhen University 1st Affiliated Hospital, Shenzhen Second People’s Hospital, Shenzhen, Guangdong, China; Banner Alzheimer's Institute, UNITED STATES

## Abstract

Detecting early morphological changes in the brain and making early diagnosis are important for Alzheimer’s disease (AD). High resolution magnetic resonance imaging can be used to help diagnosis and prediction of the disease. In this paper, we proposed a machine learning method to discriminate patients with AD or mild cognitive impairment (MCI) from healthy elderly and to predict the AD conversion in MCI patients by computing and analyzing the regional morphological differences of brain between groups. Distance between each pair of subjects was quantified from a symmetric diffeomorphic registration, followed by an embedding algorithm and a learning approach for classification. The proposed method obtained accuracy of 96.5% in differentiating mild AD from healthy elderly with the whole-brain gray matter or temporal lobe as region of interest (ROI), 91.74% in differentiating progressive MCI from healthy elderly and 88.99% in classifying progressive MCI versus stable MCI with amygdala or hippocampus as ROI. This deformation-based method has made full use of the pair-wise macroscopic shape difference between groups and consequently increased the power for discrimination.

## Introduction

Alzheimer disease (AD), the most common form of dementia, is known for the unresolved etiology and pathophysiology. Neurofibrillary tangle, plaque buildup and tissue loss in the brain parenchyma [1, 2] suggest the progressive degenerative nature of the disease. Early detection of AD at the preclinical stage is of great importance in terms of patient management. Since the earliest symptoms of AD, such as short-term memory loss and paranoid suspicion, are often mistaken as related to aging and stress, or are confused with symptoms resulted from other brain disorders, it remains challenging to predict the disease onset and the dynamic of AD in the scenario of dementia till it manifests severe cognitive impairment with typical neuroimaging signs.

AD is usually diagnosed clinically from the patient history and cognitive impairment testing [3]. Interviews with family members and caregivers are also utilized in the assessment of the disease [4]. The diagnosis based on neuropsychological scale requires rich clinical experience of physicians, and as a result it is subjective and less repeatable. Moreover, it is more challenging to identify patients suffering from AD at a prodromal stage, named mild cognitive impairment (MCI), as these subjects have cognitive impairments beyond that expected for their age and education but do not meet neuropathological criteria for AD. Neuroimaging, especially the high resolution magnetic resonance imaging (MRI), was recommended in more precise research criteria for prediction or early diagnosis of AD [5]. The structural MR images provide additional information about abnormal tissue atrophy or other abnormal biomarkers that can be sensitively detected at the early stage of the disease, and therefore automatic image-analysis methods are desired to help diagnose the illness before irreversible neuronal loss has set in, or to help detect brain changes between patients who may convert and may not convert to AD [6].

To this end, many algorithms on distinguishing AD or MCI have been proposed, varying from conceptually simple measurement of volumes or mathematically complex description of shape difference in a priori regions of interest (ROI) [7–13], to voxel-wise modeling of tissue density changes on the whole brain region, e.g. voxel-wise morphometry [11, 14–18]. There has been interest in machine learning and computer-aided diagnostics in the field of medical imaging, where a machine learning algorithm is trained to produce a desired output from a set of input training data such as features obtained from voxel intensity, tissue density or shape descriptor. Machine learning diagnostics can be also divided into ROI based and whole-brain based methods. ROI based algorithms always focus on the medial temporal structures of the brain, including the hippocampus and entorhinal cortex. In the work of Chupin et al. [19], Gutman et al. [20] and Gerardin et al. [21], support vector machine (SVM) were used for classification of AD or MCI subjects with hippocampal volume or shape as features. Another study has compared the linear discriminant analysis (LDA) and SVM for MCI classification and prediction based on hippocampal volume [22]. The entorhinal cortical thickness and modified tissue density in amygdala, parahippocampal gyrus have also been used as features in AD and MCI discrimination [23, 24]. ROI based analyses typically do not make use of all the available information contained in the whole brain, and require a priori decisions concerning which structures to assess. Atrophy in the inferior-lateral temporal lobes, cingulate gyrus, and in the parietal and frontal lobes has also been reported [25, 26]. Whether hippocampus, medial temporal lobe, or other ROIs would be a better choice for discrimination or prediction of AD is still controversial. Algorithms that extracted features from wider or cohort-adaptive brain regions have been proposed [27–32]. Kloppel et al. [33] developed a supervised method using linear SVM to group the gray matter segment of T1-weighted MR images on a high dimensional space, treating voxels as coordinates and intensity value at each voxel as their location. Aguilar et al. [34] explored the classification performance of orthogonal projections to latent structures (OPLS), decision trees, artificial neural networks (ANN), and SVM based on 10 features selected from 23 volumetric and 34 cortical thickness variables. Beheshti et al. [35] combined voxel-based morphometry and Fisher Criterion for feature selection and reduction over the entire brain, followed by SVM for classification. The whole-brain techniques have shown high discriminative power for individual diagnoses.

In this paper, we proposed a deformation-based machine learning method that quantified deformation field between subjects as distance and projected each subject onto a low dimensional Euclidean space in which a machine learning algorithm was applied to classify groups of mild AD versus normal elderly subjects, progressive MCI versus normal elderly, and stable MCI versus progressive MCI, aiming for individual patient diagnosis and predicting the conversion to AD in MCI patients.

## Materials and methods

### Data and subjects

Data used in the study were obtained from the Alzheimer’s Disease Neuroimaging Initiative (ADNI) database (http://adni.loni.usc.edu/). The ADNI was launched in 2003 by the National Institute on Aging (NIA), the National Institute of Biomedical Imaging and Bioengineering (NIBIB), the Food and Drug Administration (FDA), private pharmaceutical companies and non-profit organizations, as a $60 million, 5-year public-private partnership. The primary goal of ADNI has been to test whether serial magnetic resonance imaging (MRI), positron emission tomography (PET), other biological markers, and clinical and neuropsychological assessment can be combined to measure the progression of mild cognitive impairment (MCI) and early Alzheimer’s disease (AD). ADNI is the result of efforts of many investigators from a broad range of academic institutions and private corporations, and subjects have been recruited from over 50 sites across the U.S. and Canada. The ADNI study was approved by IRB of all participating sites. Written informed consent was provided by all subjects and if applicable, their legal representatives. For up-to-date information, see www.adni-info.org.

Data from a total of 427 subjects was retrieved from the ADNI database for whom preprocessed images and FreeSurfer post-processed images were available. The subjects were categorized into groups of normal elderly controls (NC) (n = 135, aged 76.19±5.48), stable MCI subjects (sMCI) (n = 132, aged 75.25±7.27) who had not converted to AD within 36 months, progressive MCI subjects (pMCI) (n = 95, aged 75.1±7.05) who had converted to AD 36 months after their baseline visit, and mild AD patients (n = 65, aged 75.58±8.39). The criteria used to characterize and to track a patient’s level of impairment were as follows: normal controls had a CDR (Clinical Dementia Rating) of 0 and MMSE (Mini-Mental State Examination) score between 24 and 30, MCI subjects had a CDR of 0.5 and MMSE score between 22 and 30, and mild AD patients had a CDR of 1 and MMSE score between 20 and 26 at the baseline test. Detailed demographic information of the studied population was listed in Table 1.

**Table 1:** Demographic information of the studied population.

Groups	Number	Gender (M/F)	Age (mean±std)	Baseline CDR	Baseline MMSE (mean±std)
NC	135	64/71	76.19±5.48	0	29.15±1.05
sMCI	132	90/42	75.25±7.27	0.5	27.03±1.89
pMCI	95	59/36	75.1±7.05	0.5	26.81±1.96*
AD	65	33/32	75.58±8.39	1	22.71±2.06

NC: Normal Controls; sMCI: stable Mild Cognitive Impairment; pMCI: progressive Mild Cognitive Impairment; AD: Alzheimer disease.

*An outlier with MMSE of 21 was excluded in calculation.

**Fig 1:**
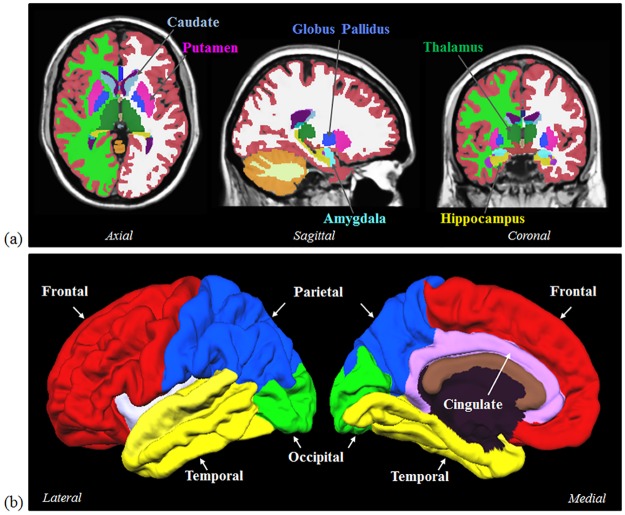
(a) Six subcortical structures including caudate, putamen, globus pallidus, hippocampus, amygdala, and thalamus were selected as ROIs. (b) Five cerebral cortical regions including frontal, parietal, occipital, temporal, and cingulate were also selected as ROIs.

The baseline 3D T1-weighted image of each subject was used for segmentation and classification using FreeSurfer (http://surfer.nmr.mgh.harvard.edu/). In this study, we have only chosen the subjects with provided FreeSurfer processing in the database to exclude segmentation variance due to different software-related settings and standard of quality control. The FreeSurfer processing in ADNI was performed by the team from Center for Imaging of Neurodegenerative Diseases, UCSF. The analysis was completed using Version 4.3 and quality control was conducted with both global and regional assessment, including the checking of skullstripped brainmask, surface segmentation and generation. The classical pipeline (*recon-all*) was conducted to each image, including intensity normalization, skull stripping, alignment to a standard space, tissue partition, surface reconstruction and inflation, spherical mapping to standard coordinate system, as well as parcellation of cerebral cortex [36–40]. The whole-brain gray matter (GM), whole-brain white matter (WM), frontal lobe, parietal lobe, occipital lobe, temporal lobe, cingulate cortex, as well as amygdala, hippocampus, caudate, putamen, globus pallidus, and thalamus were selected as regions of interest (ROI) (Fig 1).

### Registration and distance metric

Images of each subject were affinely aligned to the MNI space using FSL *flirt* (https://fsl.fmrib.ox.ac.uk/fsl/fslwiki/FLIRT) prior to deformable registration, to remove differences in subject positioning to detect true differences in shape. The symmetric log-domain diffeomorphic demons algorithm was used for the deformable registration, whose output deformation field is invertible and symmetric with respect to the order of the inputs [41]. The algorithm defines a smooth and continuous mapping *ϕ*(.) that best aligns two images *I*_0_(.) and *I*_1_(.). The global energy function of diffeomorphic demons is

$$E_{diffeo}(I_{0},I_{1},\phi ,u)=\|I_{0}-I_{1}\circ (\phi \circ \text{exp}(u))\|+\|u{\|}^{2},$$ (1)

where ***u*** is the smooth update field, *ϕ* denotes a warping operation. The optimization is performed within the space of diffeomorphisms using updates of the form *ϕ* ∘ exp(***u***). If *ϕ* is also represented as an exponential of a smooth velocity field ***v***, i.e. *ϕ* = exp(***v***), then the diffeomorphic demons is extended to represent the complete spatial transformation in the log domain. Thus the algorithm is called the log-domain diffeomorphic demons. The algorithm defines the updating rule to be

ϕ=exp(v)←exp(Z(v,u))≈exp(v)∘exp(u)=ϕ∘exp(u). (2)

where *Z*(***v***, ***u***) is a velocity field.

The log-domain diffeomorphic demons registration has a symmetric (or inverse-consistent) extension by symmetrizing the energy function

$$\phi_{opt}=\underset{\phi }{\text{arg min}}(E(I_{0},I_{1},\phi )+E(I_{1},I_{0},\phi^{-1})).$$ (3)

After registration, the algorithm provides not only the deformation field *ϕ*, but also the logarithm of the diffeomorphism, ***v*** = log(*ϕ*), which can be directly used in computational anatomical analysis. More details about the symmetric log-domain diffeomorphic demons registration were introduced in the paper of Vercauteren et al. [41].

To compute the distance between images, the Riemannian distance was defined [42]. For each pair of images {*I*_*j*_, *I*_*k*_}, the symmetric log-domain diffeomorphic demons algorithm calculated a mapping *ϕ* from *I*_*k*_ to *I*_*j*_, a velocity field ***v*** = log(*ϕ*) (that is, *ϕ* = exp(***v***)), and an inverse mapping *ϕ*^−1^ = exp(−***v***) from *I*_*j*_ back to *I*_*k*_. The following equation was used to compute the Riemannian distance between *I*_*j*_ and *I*_*k*_:

$$\begin{array}{ll} dist(I_{j},I_{k}) & =dist(Id,\phi_{ROI})=dist(\phi_{ROI}{}^{-1},Id)\\ & =\left\|\text{log}(Id^{-1}\phi_{ROI})\right\|=\left\|\text{log}[{\left(\phi_{ROI}{}^{-1}\right)}^{-1}Id]\right\|\\ & =\left\|\text{log}(\phi_{ROI})\right\|=\left\|\text{log}[{\left(\phi_{ROI}{}^{-1}\right)}^{-1}]\right\|\\ & =\left\|v_{ROI}\right\|=\frac{\left\|v_{ROI_{j}}\right\|+\left\|{\left(-v\right)}_{ROI_{k}}\right\|}{2} \end{array}.$$ (4)

where *Id* denotes an identity transformation. In the above equation, *ϕ*_*ROI*_ can be either a diffeomorphism of the whole brain or a sub-field of any segmented region of the brain. $v_{ROI_{j}}$ and $v_{ROI_{k}}$ represent the log-domain diffeomorphism of the specific ROI in *I*_*j*_ and *I*_*k*_, respectively. For example, the specific ROI can be the whole-brain gray matter (GM) or white matter (WM), cortical lobes, hippocampus or other subcortical structures.

### Embedding algorithm

A distance matrix was constructed after the distance between each pair of subjects was calculated. The embedding algorithm projected all the labeled images onto a low-dimensional space with this distance matrix and a discrimination hyperplane will be obtained by training the labeled subjects on the embedded space. To classify a new unlabeled image, an out-of-sample extension of embedding algorithms was used to project the new subject onto the constructed embedded space.

The metric multi-dimensional scaling (MDS) algorithm was applied for embedding. The idea of metric MDS is to transform the distance matrix into a cross-product matrix and then to find its eigen-decomposition which gives a principal component analysis (PCA). Let *S*_*i*_ be the *i*-th row sum of the distance matrix *D*, *S*_*i*_ = Σ_*j*_*D*_*ij*_. The cross-product matrix is obtained by using the “double-centering” formula:

$${\tilde{D}}_{ij}=-\frac{1}{2}(D_{ij}-\frac{1}{n}S_{i}-\frac{1}{n}S_{j}+\frac{1}{n^{2}}\sum_{m}S_{m}).$$ (5)

The embedding *e*_*im*_ of subject *x*_*i*_ is $\sqrt{\lambda_{m}}v_{im}$, *m* = {1,…,*M*}, where *λ*_*m*_ denotes the *m*-th principal eigenvalue and *v*_*im*_ denotes the *i*-th element of the *m*-th principal eigenvector.

To calculate the embedding coordinate of a new point, define the kernel function $\tilde{K}$ yielding the symmetric matrix $\tilde{M}$ on the dataset $I=\{x_{1},\ldots ,x_{n}\}$, with *x*_*i*_ sampled from an unknown distribution with density *p*:

$$\tilde{K}(a,b)=-\frac{1}{2}(d^{2}(a,b)-E_{x}[d^{2}(x,b)]-E_{x}[d^{2}(a,x\prime )]+E_{x,x\prime }[d^{2}(x,x\prime )]),$$ (6)

where *d*(*a*, *b*) is the original distance and the expectations *E* are taken over the training data I. Let (*v*_*l*_, *λ*_*l*_) be an (*eigenvector*, *eigenvalue*) pair that solves $\tilde{M}v_{l}=\lambda_{l}v_{l}$ and *e*_*l*_ denotes the embedding associated with the new point *x*. Then

$$e_{l}(x)=\frac{1}{\sqrt{\lambda_{l}}}\sum_{i=1}^{n}v_{li}\tilde{K}(x,x_{i}).$$ (7)

Readers can refer to the work of Bengio et al. for algorithm details and proof [43]. In this study, subjects were all projected onto an ${\mathbb{R}}^{3}$ space for classification.

### Classification

SVM with a linear kernel which was implemented using matlab ‘libsvm’ toolbox (http://www.csie.ntu.edu.tw/~cjlin/libsvm/), was applied on the embedded space to classify subjects. The C-SVM model was chosen, and the cost parameter C was fixed as 1 in all experiments. The *k*-fold cross validation was adopted to estimate the classification performance. The subjects were randomly partitioned into *k* “equal” sized subgroups. In this study, as the number of subjects in each group was unequal and may not be evenly divided by *k*, some subgroups may have one or two more subjects in practice. Of the *k* subgroups, a single subgroup was used as the validation data and the remaining *k*-1 subgroups were used as training data. The process was repeated for *k* times and *k* was set as 10 in this study. Classification sensitivity, specificity, and accuracy were then calculated. The receiver operating characteristics (ROC) curve was plotted and areas under ROC curve (AUC) was measured.

## Results

No significant differences on age were found between each pair of groups using the Student’s *t* test. For the baseline MMSE score, no significant difference was found only between sMCI and pMCI subjects.

**Fig 2:**
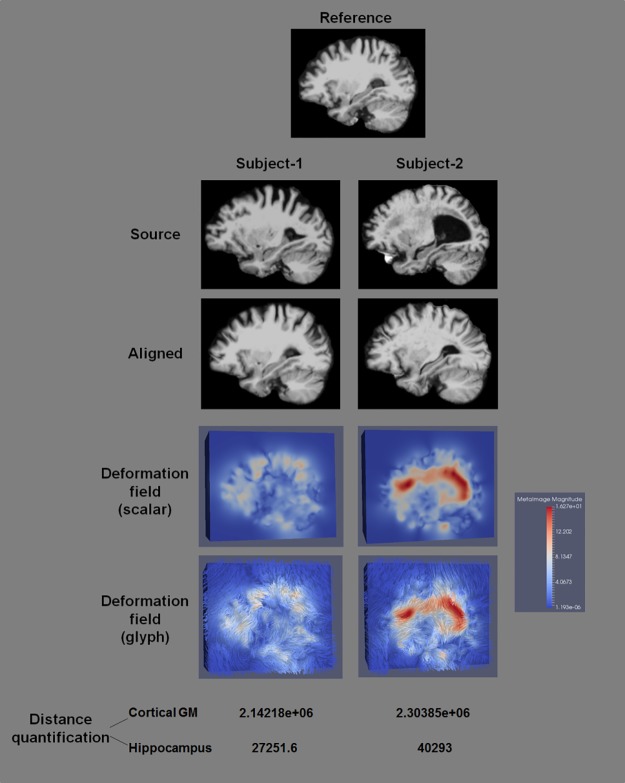
Comparison between different subjects in their deformable registration, deformation fields, and quantified distances. The symmetric log-domain diffeomorphic demons registration well aligned the reference and source images, providing informative deformation fields that accurately reflect morphological difference between subjects.

The deformable registration and distance quantification results of two pairs of subjects were shown in Fig 2, where the same reference was used. Images before and after registration, deformation fields, and quantified ROI-specific Riemannian distances for the two source subjects were shown. It was observed that the reference and source images were considerably well aligned using the symmetric log-domain diffeomorphic demons registration. The deformation from the subject who is more morphologically different from the reference was notably larger than that from the other subject. Consequently, the difference was manifest in the quantified distances.

**Fig 3:**
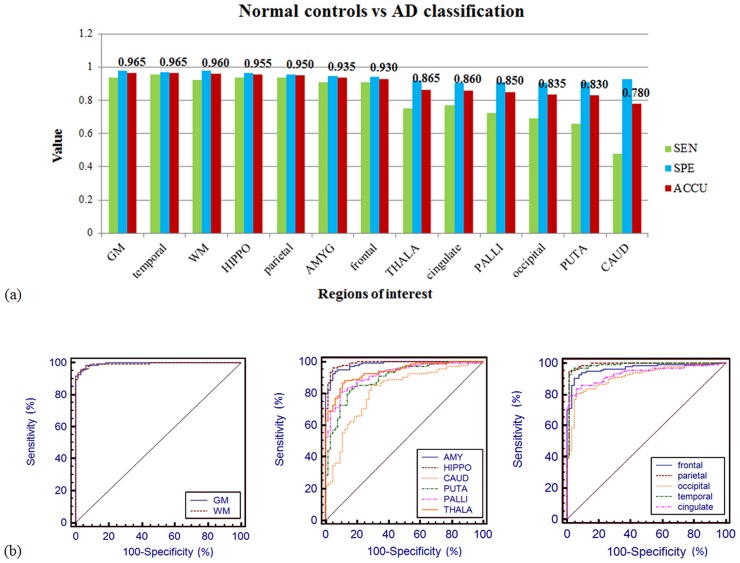
(a) Classification sensitivity (green), specificity (blue), and accuracy (red) of normal elderly controls versus AD patients with different ROIs. The highest accuracy (96.5%) was achieved using the whole-brain gray matter as ROI with 93.85% sensitivity and 97.78% specificity. The algorithm obtained high sensitivity and specificity (>90%) with half of the ROIs. (b) The ROC curve of the prediction accuracy between normal controls versus AD. The AUCs were larger than 0.98 for the whole-brain gray matter and white matter (left), amygdala and hippocampus (middle), parietal and temporal lobes (right).

Classification results for differentiating normal elderly controls and AD patients were summarized in Table 2 and Fig 3. Using the whole-brain gray matter as ROI, the highest classification accuracy was 96.5% with a sensitivity of 93.85%, specificity of 97.78% and AUC of 0.995. In addition, using the other six ROIs including temporal lobe, whole-brain white matter, hippocampus, parietal lobe, amygdala, and frontal lobe, the algorithm achieved high sensitivity and specificity above 90% (AUCs>0.96). The worst performance resulted from caudate, where the sensitivity was substantially lower in discrimination.

**Table 2:** Classification results of normal elderly controls vs AD.

ROIs		SEN	SPE	PPV	NPV	ACCU	AUC	Ranking
Whole brain	GM	93.85%	97.78%	95.31%	97.06%	96.50%	0.995	1
	WM	92.31%	97.78%	95.24%	96.35%	96.00%	0.993	3
Subcortical structures	AMYG	90.77%	94.82%	89.39%	95.52%	93.50%	0.983	6
	HIPPO	93.85%	96.30%	92.42%	97.02%	95.50%	0.989	4
	CAUD	47.69%	92.59%	75.61%	78.62%	78.00%	0.815	13
	PUTA	66.15%	91.11%	78.18%	84.83%	83.00%	0.896	12
	PALLI	72.31%	91.11%	79.66%	87.23%	85.00%	0.920	10
	THALA	75.39%	91.85%	81.67%	88.57%	86.50%	0.937	8
Cortical lobes	Frontal	90.77%	94.07%	88.06%	95.49%	93.00%	0.966	7
	Parietal	93.85%	95.56%	91.05%	96.99%	95.00%	0.987	5
	Occipital	69.23%	90.37%	77.59%	85.92%	83.50%	0.914	11
	Temporal	95.39%	97.04%	93.94%	97.76%	96.50%	0.984	2
	Cingulate	76.92%	90.37%	79.37%	89.05%	86.00%	0.936	9

SEN: sensitivity; SPE: specificity; PPV: positive predictive value; NPV: negative predictive value; ACCU: accuracy; AUC: area under ROC curve. GM: whole-brain gray matter; WM: whole-brain white matter; AMYG: amygdala; HIPPO: hippocampus; CAUD: caudate; PUTA: putamen; PALLI: globus pallidus; THALA: thalamus.

**Fig 4:**
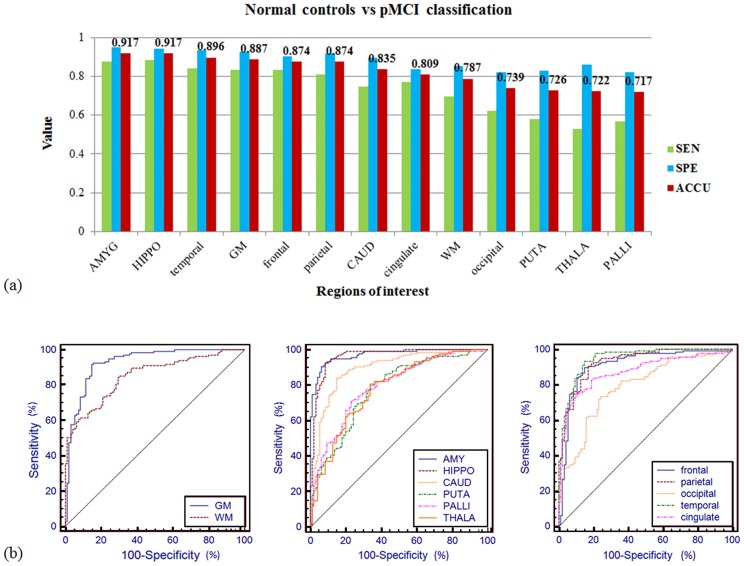
(a) Classification sensitivity (green), specificity (blue), and accuracy (red) of normal elderly controls versus progressive MCI subjects with different ROIs. Using the amygdala, and hippocampus as ROI, the algorithm obtained classification accuracy of 91.74%. With the other six ROIs (temporal, GM, frontal, parietal, caudate, and cingulate), the accuracy exceeded 80%. (b) The ROC curve of the prediction accuracy between normal controls versus progressive MCI. The AUC reached up to 0.971 for amygdala (middle-blue curve), and was larger than 0.91 for hippocampus (0.963), temporal lobe (0.947), the whole-brain gray matter (0.928), parietal lobe (0.928), and frontal lobe (0.912).

Classification results for normal elderly controls versus progressive MCI subjects were summarized in Table 3 and Fig 4. Our method obtained accuracy of 91.74% with amygdala (87.37% sensitivity, 94.82% specificity, 0.971 AUC) and hippocampus (88.42% sensitivity, 94.07% specificity, 0.963 AUC) as ROI respectively. The sensitivity and specificity were higher than 80% for the other four ROIs including temporal lobe (84.21% and 93.33%), whole-brain gray matter (83.16% and 92.59%), frontal lobe (83.16% and 90.37%), and parietal lobe (81.05% and 91.85%). The sensitivity values were low for the occipital lobe, putamen, thalamus, and globus pallidus, which resulted in lower classification accuracy from 71.74% to 73.91% for these ROIs.

**Table 3:** Classification results of normal elderly controls vs pMCI.

ROIs		SEN	SPE	PPV	NPV	ACCU	AUC	Ranking
Whole brain	GM	83.16%	92.59%	88.76%	88.65%	88.70%	0.928	4
	WM	69.47%	85.19%	76.74%	79.86%	78.70%	0.851	9
Subcortical structures	AMYG	87.37%	94.82%	92.22%	91.43%	91.74%	0.971	1
	HIPPO	88.42%	94.07%	91.30%	92.03%	91.74%	0.963	2
	CAUD	74.74%	89.63%	83.53%	83.45%	83.48%	0.894	7
	PUTA	57.90%	82.96%	70.51%	73.68%	72.61%	0.777	11
	PALLI	56.84%	82.22%	69.23%	73.03%	71.74%	0.793	13
	THALA	52.63%	85.93%	72.46%	72.05%	72.17%	0.778	12
Cortical lobes	Frontal	83.16%	90.37%	85.87%	88.41%	87.39%	0.912	5
	Parietal	81.05%	91.85%	87.50%	87.32%	87.39%	0.928	6
	Occipital	62.11%	82.22%	71.08%	75.51%	73.91%	0.787	10
	Temporal	84.21%	93.33%	89.89%	89.36%	89.57%	0.947	3
	Cingulate	76.84%	83.70%	76.84%	83.70%	80.87%	0.873	8

SEN: sensitivity; SPE: specificity; PPV: positive predictive value; NPV: negative predictive value; ACCU: accuracy; AUC: area under ROC curve. GM: whole-brain gray matter; WM: whole-brain white matter; AMYG: amygdala; HIPPO: hippocampus; CAUD: caudate; PUTA: putamen; PALLI: globus pallidus; THALA: thalamus.

**Fig 5:**
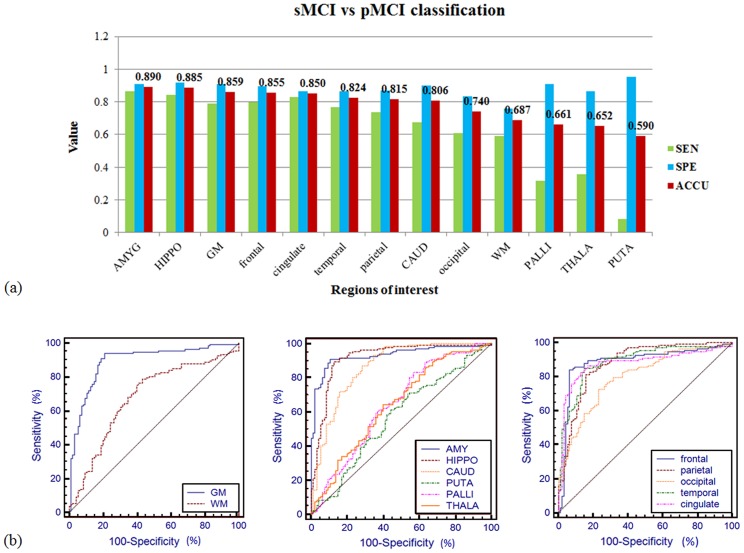
(a) Classification sensitivity (green), specificity (blue), and accuracy (red) of stable MCI versus progressive MCI subjects with different ROIs. High ranked ROIs included amygdala, hippocampus, the whole-brain gray matter, frontal lobe, and cingulate cortex, with which classification accuracy exceeded 85%. Sensitivity for globus pallidus, thalamus, and putamen was substantially low which resulted in bad performance in discrimination. (b) The ROC curve of the prediction accuracy between stable MCI versus progressive MCI. The AUC reached up to 0.932 for amygdala (middle-blue curve), and 0.918 for hippocampus (middle-maroon curve).

Classification results for stable MCI versus progressive MCI subjects were summarized in Table 4 and Fig 5. As in differentiating normal controls and pMCI subjects, amygdala and hippocampus remained the top two ROIs with which the method obtained the highest classification accuracy of 89% (86.32% sensitivity, 90.91% specificity, 0.932 AUC) and 88.5% (84.21% sensitivity, 91.67% specificity, 0.918 AUC) respectively. The algorithm also performed well when using the whole-brain gray matter, frontal lobe, and cingulate cortex as ROI, achieving accuracy over 85% (AUCs>0.875). For the globus pallidus, thalamus, and putamen, we obtained high specificity but significantly lower sensitivity, resulting in classification accuracy lower than 67%.

**Table 4:** Classification results of sMCI vs pMCI.

ROIs		SEN	SPE	PPV	NPV	ACCU	AUC	Ranking
Whole brain	GM	78.95%	90.91%	86.21%	85.71%	85.90%	0.892	3
	WM	58.95%	75.76%	63.64%	71.94%	68.72%	0.679	10
Subcortical structures	AMYG	86.32%	90.91%	87.23%	90.23%	88.99%	0.932	1
	HIPPO	84.21%	91.67%	87.91%	88.97%	88.55%	0.918	2
	CAUD	67.37%	90.15%	83.12%	79.33%	80.62%	0.864	8
	PUTA	8.42%	95.46%	57.14%	59.16%	59.03%	0.571	13
	PALLI	31.58%	90.91%	71.43%	64.87%	66.08%	0.643	11
	THALA	35.79%	86.36%	65.39%	65.14%	65.20%	0.642	12
Cortical lobes	Frontal	80.00%	89.39%	84.44%	86.13%	85.46%	0.886	4
	Parietal	73.68%	87.12%	80.46%	82.14%	81.50%	0.88	7
	Occipital	61.05%	83.33%	72.50%	74.83%	74.01%	0.798	9
	Temporal	76.84%	86.36%	80.22%	83.82%	82.38%	0.889	6
	Cingulate	83.16%	86.36%	81.44%	87.69%	85.02%	0.876	5

SEN: sensitivity; SPE: specificity; PPV: positive predictive value; NPV: negative predictive value; ACCU: accuracy; AUC: area under ROC curve. GM: whole-brain gray matter; WM: whole-brain white matter; AMYG: amygdala; HIPPO: hippocampus; CAUD: caudate; PUTA: putamen; PALLI: globus pallidus; THALA: thalamus.

To summarize and compare the classification performance of each ROI, we calculated the mean accuracy for each ROI over the three experiments (Table 5). Hippocampus and amygdala were ranked the top two ROIs with excellent performance for all testing. Gray matter and its subdivisions also got high rankings except for the occipital lobe, followed by the white matter and other subcortical structures.

**Table 5:** Classification performance comparison for different ROIs.

Ranking	ROIs	Accuracy			Mean Accuracy
		NC vs AD	NC vs pMCI	sMCI vs pMCI	
1	HIPPO	95.50%	91.74%	88.55%	91.93%
2	AMYG	93.50%	91.74%	88.99%	91.41%
3	GM	96.50%	88.70%	85.90%	90.37%
4	temporal	96.50%	89.57%	82.38%	89.48%
5	frontal	93.00%	87.39%	85.46%	88.62%
6	parietal	95.00%	87.39%	81.50%	87.96%
7	cingulate	86.00%	80.87%	85.02%	83.96%
8	WM	96.00%	78.70%	68.72%	81.14%
9	CAUD	78.00%	83.48%	80.62%	80.70%
10	occipital	83.50%	73.91%	74.01%	77.14%
11	THALA	86.50%	72.17%	65.20%	74.62%
12	PALLI	85.00%	71.74%	66.08%	74.27%
13	PUTA	83.00%	72.61%	59.03%	71.55%

GM: whole-brain gray matter; WM: whole-brain white matter; AMYG: amygdala; HIPPO: hippocampus; CAUD: caudate; PUTA: putamen; PALLI: globus pallidus; THALA: thalamus.

## Discussion

### Classification performance compared with existing algorithms

A lot of algorithms have been proposed for early diagnosis of AD with accuracy ranging from 75% to 96% [44–48]. Kloppel et al. considered the voxels of tissue probability maps of the whole brain or volumes of interest (VOI) as features in the classification, obtaining accuracy of 95.6% to discriminate normal controls and AD [33]. In recent work, Beheshti et al. selected the regions with significant difference between groups as VOIs and considered each voxel in the VOIs as a feature, followed by a feature selection step [35]. They obtained 96.32% accuracy between controls and AD. In this work, we observed a classification rate of 96.5% using the whole-brain gray matter as ROI with an AUC of 0.995. For five ROIs, the classification accuracy exceeded 95% indicating that global morphological changes have occurred in mild AD patients and that mild AD is much distinguishable from healthy controls.

By contrast, the brain shape difference between healthy elderly and MCI subjects is smaller, which therefore increases difficulty for discrimination. Fan et al. proposed a method that considered the tissue density from pathology-adaptive anatomical parcellation as features and obtained classification accuracy of 81.8% [48]. Chupin et al. used hippocampal volume to discriminate between elderly controls and progressive MCI who had developed AD in 18 months and obtained 71% accuracy [19]. Our proposed algorithm manifested outstanding performance in the testing, where 91.74% accuracy (0.971 AUC) was obtained to classify MCI who had developed AD at 36 months follow-up. For the ROIs of amygdala, hippocampus, temporal lobe, the whole-brain gray matter, frontal lobe, and parietal lobe, the algorithm obtained AUC values all higher than 0.9.

To distinguish progressive MCI from stable MCI, which is important for prediction of conversion in MCI subjects, is challenging in the MRI-based classification. An algorithm based on hippocampal volume measurement obtained accuracy of 67% [19]. Normalized thickness index in specific cortical regions was considered as features in another algorithm proposed by Querbes et al. [49], where 76% accuracy was obtained to classify MCI converters for the 24-month period. Lillemark et al. reported an classification accuracy of 76.6% using the region-based surface connectivity as features for grouping MCI subjects who had developed AD at 12-month follow-up [50]. Westman et al. [45] and Aguilar et al. [34] collected multiple surface and volumetric indices via FreeSurfer processing and applied multivariate models for discrimination respectively. Westman et al. obtained 75.9% accuracy for MCIs with conversion at 18 months follow-up while Aguilar et al. obtained 86% accuracy for MCIs with conversion at 12 months follow-up. Using the proposed method, we obtained an overall accuracy of 88.99% (0.932 AUC) to classify MCI patients who had progressed to AD after 36 months of baseline visit. Algorithm comparison was summarized in Table 6.

**Table 6:** Comparison between the proposed method and existing methods.

Methods	Sample size	Type of validation	NC vs AD		NC vs MCI		sMCI vs pMCI		Conversion time after baseline
			ACCU	ROI	ACCU	ROI	ACCU	ROI	
Kloppel [33]	67 AD, 91 HC	Leave one out and random subsampling	95.6%	Whole brain	–	–	–	–	–
Magnin [51]	16 AD, 22HC	Bootstrap subsampling	94.5%	Whole brain	–	–	–	–	–
Beheshti [35]	68 AD, 68 HC	10-fold	96.32%	GM	–	–	–	–	–
Fan [48]	56 AD, 88 MCI, 66 HC	Leave one out	94.3%	Pathology-adaptive parcellation	81.8%	Pathology-adaptive parcellation	–	–	–
Chupin [19]	122 AD, 65 pMCI, 121 sMCI, 128HC	Bootstrap subsampling	80%	Hippocampus	74%	Hippocampus	67%	Hippocampus	≤18 months
Lillemark [50]	114 AD, 240 MCI, 170 HC	Leave one out	0.877 (AUC)	Cerebral cortex, WM; cerebellum cortex, WM; inf lateral ventricle; thala., etc.	0.785 (AUC)	Cerebral cortex, WM; cerebellum cortex, WM; inf lateral ventricle; thala., etc.	0.599 (AUC)	Cerebral cortex, WM; cerebellum cortex, WM; inf lateral ventricle; thala., etc.	12 months
Westman [45]	187 AD, 87 pMCI, 200 sMCI, 225 HC	7-fold	91.8%	34 cortical parcellation and 21 subcortical regions by FreeSurfer	–	–	75.9%	34 cortical parcellation and 21 subcortical regions by FreeSurfer	18 months
Aguilar [34]	116 AD, 21 pMCI, 98 sMCI, 110 HC	10-fold	85%	34 cortical parcellation and 50 subcortical regions by FreeSurfer	–	–	86%	34 cortical parcellation and 50 subcortical regions by FreeSurfer	12 months
Querbes [49]	72 pMCI, 50 sMCI	10-fold	–	–	–	–	76%	Right medial temporal, left lateral temporal, right posterior cingulate	24 months
**Proposed method**	**65 AD, 95 pMCI, 132 sMCI, 135 HC**	**10-fold**	**96.5%**	**Whole brain GM**	**91.73%**	**Amygdala or hippocampus**	**88.99%**	**Amygdala or hippocampus**	**36 months**

The proposed algorithm developed a new strategy that quantified the deformation field to represent shape difference between subjects rather than comparing the tissue density or surface/volumetric indices. This deformation-based method characterized the macroscopic differences in brain anatomy which were discarded in most of the existing approaches at the spatial normalization step. The quantified deformation was then used to denote dissimilarity between subjects and a distance matrix was constructed. The MDS algorithm used in the study was guaranteed to recover the true dimensionality and geometric structure of manifolds in which each subject represented as an element [52]. Finally MDS constructed an embedding of the data in a low-dimensional Euclidean space that best preserved the manifold’s estimated intrinsic geometry. The advantage of this algorithm may due to the as much information it used in dimensional reduction for spatially representing the similarity relationships between subjects, by computing the pair-wise registration instead of aligning subjects to an atlas or a constructed template, resulting in more informative embedding and consequently an enhanced power to discriminate between different populations.

### Prediction of AD conversion in MCI patients

Identifying MCI patients at high risk for conversion to AD is crucial for the effective treatment of the disease. Over the past decade, numerous biomarkers have been proposed for prediction of AD-conversion in MCI patients [19, 34, 45, 49, 50, 53–58]. Cognitive performance data including the Spatial Pattern of Abnormalities for Recognition of Early AD (SPARE-AD) index, AD Assessment Scale-Cognitive (ADAS-Cog) subscale, or composite cognitive scores were introduced to assess AD conversion. However, the accuracy is not satisfactory with a classification rate around 65% [44]. Combining cognitive measures with MRI and age information, the discrimination rate has risen to 82% [57]. Cerebrospinal fluid (CSF) tau and Aβ42 measures have been also proposed as potential predictors of risk for developing AD [59]. Integrating CSF biomarkers together with MRI patterns resulted in accuracy of 62% [53]. When further including positron emission tomography (PET) data and routine clinical tests, the predicting accuracy has increased to 72% [55].

Compared to previous studies using ADNI database, the proposed algorithm based on quantification of MRI deformation demonstrated a promising strategy for predicting MCI-to-AD conversion 3 years in advance with accuracy of 88.99% and AUC of 0.932, which are the highest rates ever reported to the best of our knowledge. If MRI can provide sufficient information for good prediction using a robust algorithm, the use of CSF and PET biomarkers can be avoided as the former requires lumbar puncture which is invasive and painful for patients and the latter suffers its high cost and radiation exposure [60].

### Selection of regions of interest for classification

Global and regional cerebral atrophy has been reported in previous studies. Annual rates of global brain atrophy in AD are about 2–3%, compared with 0.2–0.5% in healthy controls [6, 61]. At early stage of AD progression, prominent atrophy has emerged in the medial temporal regions and the posterior cortical regions including posterior cingulate, retrosplenial, and lateral parietal cortex [62]. Medial temporal lobe atrophy, particularly of the amygdala, hippocampus, entorhinal cortex, and parahippocampal gyrus, can be observed with higher frequency in patients with AD or probable AD [63, 64]. Shape changes have also been demonstrated in the caudate, putamen, globus pallidus, and thalamus in AD [65].

Although remarkable morphological alterations were found in a certain regions in AD or prodromal AD at the group level, individual classification based on different regions in this study yielded substantially distinct results. The whole-brain gray matter and temporal lobe performed the best in distinguishing AD from normal elderly controls, while amygdala and hippocampus worked better in classifying progressive MCI versus either healthy elderly or stable MCI. This result was mostly consistent with the previous finding that significantly increased rates of hippocampal atrophy were observed in presymptomatic and mild AD, while more widespread tissue shrinkage has been shown in mild to moderate AD patients [6, 66]. Evidence have also been documented that increased oxygen extraction capacity and tissue atrophy were observed in basal ganglia and thalamus in patient with AD [65, 67]. These ROIs indeed resulted in a classification accuracy higher than 80% in discriminating AD, nevertheless much lower in classifying progressive MCI, indicating that shape changes of basal ganglia and thalamus were prominent features in AD but not yet in the prodromal stage. By an integrative comparison, we proposed that hippocampus, amygdala, the whole-brain gray matter, temporal lobe, and parietal lobe should be of higher preference for AD or MCI classification, where amygdala and hippocampus could be the leading candidate for predicting AD conversion in MCI, while occipital lobe, thalamus, globus pallidus, and putamen should be non-priority selections for early diagnosis.

## Conclusion

In this study, we proposed a deformation-based machine learning method for discrimination of AD and prediction of MCI-to-AD conversion with high resolution MRI. The proposed algorithm showed great performance on both classification and prediction of AD, with 96.5% accuracy discriminating AD from healthy elderly, 91.74% accuracy for progressive MCI versus healthy elderly, and 88.99% accuracy for progressive MCI versus stable MCI. Large deformation in hippocampus and amygdala was advantageous to differentiate progressive MCI patients, while diffusive morphological changes in the whole-brain gray matter were prominent to identify mild or moderate AD patients.

The limitation of the algorithm is that it was computational expensive. A balance between classification accuracy and computational time should be achieved in our future research. In general, MRI-based analysis can be a beneficial supplement to clinical diagnosis and prediction of AD.

## Supporting information

S1 File ADNI acknowledgement list. (PDF)

## References

[1] Braak H, Braak E. Neuropathological stageing of Alzheimer-related changes. Acta neuropathologica. 1991;82(4):239–59. Epub 1991/01/01. 1759558 10.1007/BF00308809

[2] Tiraboschi P, Hansen LA, Thal LJ, Corey-Bloom J. The importance of neuritic plaques and tangles to the development and evolution of AD. Neurology. 2004;62(11):1984–9. Epub 2004/06/09. 15184601 10.1212/01.wnl.0000129697.01779.0a

[3] Blennow K, de Leon MJ, Zetterberg H. Alzheimer's disease. Lancet (London, England). 2006;368(9533):387–403. Epub 2006/08/01. 10.1016/S0140-6736(06)69113-7 16876668

[4] Harvey PD, Moriarty PJ, Kleinman L, Coyne K, Sadowsky CH, Chen M, et al The validation of a caregiver assessment of dementia: the Dementia Severity Scale. Alzheimer disease and associated disorders. 2005;19(4):186–94. Epub 2005/12/06. 16327345 10.1097/01.wad.0000189034.43203.60

[5] Dubois B, Feldman HH, Jacova C, Dekosky ST, Barberger-Gateau P, Cummings J, et al Research criteria for the diagnosis of Alzheimer's disease: revising the NINCDS-ADRDA criteria. The Lancet Neurology. 2007;6(8):734–46. Epub 2007/07/10. 10.1016/S1474-4422(07)70178-3 17616482

[6] Fox NC, Schott JM. Imaging cerebral atrophy: normal ageing to Alzheimer's disease. Lancet (London, England). 2004;363(9406):392–4. Epub 2004/04/13. 10.1016/S0140-6736(04)15441-X 15074306

[7] Jack CR Jr., Petersen RC, Xu YC, O'Brien PC, Smith GE, Ivnik RJ, et al Prediction of AD with MRI-based hippocampal volume in mild cognitive impairment. Neurology. 1999;52(7):1397–403. Epub 1999/05/05. 10227624 10.1212/wnl.52.7.1397 PMC2730146

[8] Barnes J, Scahill RI, Boyes RG, Frost C, Lewis EB, Rossor CL, et al Differentiating AD from aging using semiautomated measurement of hippocampal atrophy rates. NeuroImage. 2004;23(2):574–81. Epub 2004/10/19. 10.1016/j.neuroimage.2004.06.028 15488407

[9] Colliot O, Chetelat G, Chupin M, Desgranges B, Magnin B, Benali H, et al Discrimination between Alzheimer disease, mild cognitive impairment, and normal aging by using automated segmentation of the hippocampus. Radiology. 2008;248(1):194–201. Epub 2008/05/07. 10.1148/radiol.2481070876 18458242

[10] Good CD, Scahill RI, Fox NC, Ashburner J, Friston KJ, Chan D, et al Automatic differentiation of anatomical patterns in the human brain: validation with studies of degenerative dementias. NeuroImage. 2002;17(1):29–46. Epub 2002/12/17. 12482066 10.1006/nimg.2002.1202

[11] Busatto GF, Garrido GE, Almeida OP, Castro CC, Camargo CH, Cid CG, et al A voxel-based morphometry study of temporal lobe gray matter reductions in Alzheimer's disease. Neurobiology of aging. 2003;24(2):221–31. Epub 2002/12/25. 12498956 10.1016/s0197-4580(02)00084-2

[12] Tapiola T, Pennanen C, Tapiola M, Tervo S, Kivipelto M, Hanninen T, et al MRI of hippocampus and entorhinal cortex in mild cognitive impairment: a follow-up study. Neurobiology of aging. 2008;29(1):31–8. Epub 2006/11/14. 10.1016/j.neurobiolaging.2006.09.007 17097769

[13] Xu Y, Jack CR Jr., O'Brien PC, Kokmen E, Smith GE, Ivnik RJ, et al Usefulness of MRI measures of entorhinal cortex versus hippocampus in AD. Neurology. 2000;54(9):1760–7. Epub 2000/05/10. 10802781 10.1212/wnl.54.9.1760

[14] Hirata Y, Matsuda H, Nemoto K, Ohnishi T, Hirao K, Yamashita F, et al Voxel-based morphometry to discriminate early Alzheimer's disease from controls. Neuroscience letters. 2005;382(3):269–74. Epub 2005/06/01. 10.1016/j.neulet.2005.03.038 15925102

[15] Bozzali M, Filippi M, Magnani G, Cercignani M, Franceschi M, Schiatti E, et al The contribution of voxel-based morphometry in staging patients with mild cognitive impairment. Neurology. 2006;67(3):453–60. Epub 2006/08/09. 10.1212/01.wnl.0000228243.56665.c2 16894107

[16] Vemuri P, Gunter JL, Senjem ML, Whitwell JL, Kantarci K, Knopman DS, et al Alzheimer's disease diagnosis in individual subjects using structural MR images: validation studies. NeuroImage. 2008;39(3):1186–97. Epub 2007/12/07. 10.1016/j.neuroimage.2007.09.073 18054253 PMC2390889

[17] Chetelat G, Landeau B, Eustache F, Mezenge F, Viader F, de la Sayette V, et al Using voxel-based morphometry to map the structural changes associated with rapid conversion in MCI: a longitudinal MRI study. NeuroImage. 2005;27(4):934–46. Epub 2005/06/28. 10.1016/j.neuroimage.2005.05.015 15979341

[18] Lao Z, Shen D, Xue Z, Karacali B, Resnick SM, Davatzikos C. Morphological classification of brains via high-dimensional shape transformations and machine learning methods. NeuroImage. 2004;21(1):46–57. Epub 2004/01/27. 14741641 10.1016/j.neuroimage.2003.09.027

[19] Chupin M, Gerardin E, Cuingnet R, Boutet C, Lemieux L, Lehericy S, et al Fully automatic hippocampus segmentation and classification in Alzheimer's disease and mild cognitive impairment applied on data from ADNI. Hippocampus. 2009;19(6):579–87. Epub 2009/05/14. 10.1002/hipo.20626 19437497 PMC2837195

[20] Gutman B, Wang Y, Morra J, Toga AW, Thompson PM. Disease classification with hippocampal shape invariants. Hippocampus. 2009;19(6):572–8. Epub 2009/05/14. 10.1002/hipo.20627 19437498 PMC3113700

[21] Gerardin E, Chetelat G, Chupin M, Cuingnet R, Desgranges B, Kim HS, et al Multidimensional classification of hippocampal shape features discriminates Alzheimer's disease and mild cognitive impairment from normal aging. NeuroImage. 2009;47(4):1476–86. Epub 2009/05/26. 10.1016/j.neuroimage.2009.05.036 19463957 PMC3001345

[22] Wolz R, Julkunen V, Koikkalainen J, Niskanen E, Zhang DP, Rueckert D, et al Multi-Method Analysis of MRI Images in Early Diagnostics of Alzheimer's Disease. PLoS One. 2011;6(10):e25446 10.1371/journal.pone.0025446 22022397 PMC3192759

[23] Ewers M, Walsh C, Trojanowski JQ, Shaw LM, Petersen RC, Jack CR Jr., et al Prediction of conversion from mild cognitive impairment to Alzheimer's disease dementia based upon biomarkers and neuropsychological test performance. Neurobiology of aging. 2012;33(7):1203–14. Epub 2010/12/17. 10.1016/j.neurobiolaging.2010.10.019 21159408 PMC3328615

[24] Duchesne S, Caroli A, Geroldi C, Barillot C, Frisoni GB, Collins DL. MRI-based automated computer classification of probable AD versus normal controls. IEEE transactions on medical imaging. 2008;27(4):509–20. Epub 2008/04/09. 10.1109/TMI.2007.908685 18390347

[25] Karas GB, Scheltens P, Rombouts SA, Visser PJ, van Schijndel RA, Fox NC, et al Global and local gray matter loss in mild cognitive impairment and Alzheimer's disease. NeuroImage. 2004;23(2):708–16. Epub 2004/10/19. 10.1016/j.neuroimage.2004.07.006 15488420

[26] Lerch JP, Pruessner JC, Zijdenbos A, Hampel H, Teipel SJ, Evans AC. Focal decline of cortical thickness in Alzheimer's disease identified by computational neuroanatomy. Cerebral cortex (New York, NY: 1991). 2005;15(7):995–1001. Epub 2004/11/13. 10.1093/cercor/bhh200 15537673

[27] Yang W, Lui RL, Gao JH, Chan TF, Yau ST, Sperling RA, et al Independent component analysis-based classification of Alzheimer's disease MRI data. Journal of Alzheimer's disease: JAD. 2011;24(4):775–83. Epub 2011/02/16. 10.3233/JAD-2011-101371 21321398 PMC3697832

[28] Adaszewski S, Dukart J, Kherif F, Frackowiak R, Draganski B. How early can we predict Alzheimer's disease using computational anatomy? Neurobiology of aging. 2013;34(12):2815–26. Epub 2013/07/31. 10.1016/j.neurobiolaging.2013.06.015 23890839

[29] Varol E, Gaonkar B, Erus G, Schultz R, Davatzikos C. FEATURE RANKING BASED NESTED SUPPORT VECTOR MACHINE ENSEMBLE FOR MEDICAL IMAGE CLASSIFICATION. Proceedings IEEE International Symposium on Biomedical Imaging. 2012:146–9. Epub 2012/01/01. 10.1109/ISBI.2012.6235505 23873289 PMC3715725

[30] Spulber G, Simmons A, Muehlboeck JS, Mecocci P, Vellas B, Tsolaki M, et al An MRI-based index to measure the severity of Alzheimer's disease-like structural pattern in subjects with mild cognitive impairment. Journal of internal medicine. 2013;273(4):396–409. Epub 2013/01/03. 10.1111/joim.12028 23278858 PMC3605230

[31] Koikkalainen J, Lotjonen J, Thurfjell L, Rueckert D, Waldemar G, Soininen H. Multi-template tensor-based morphometry: application to analysis of Alzheimer's disease. NeuroImage. 2011;56(3):1134–44. Epub 2011/03/23. 10.1016/j.neuroimage.2011.03.029 21419228 PMC3554792

[32] Nho K, Shen L, Kim S, Risacher SL, West JD, Foroud T, et al Automatic Prediction of Conversion from Mild Cognitive Impairment to Probable Alzheimer's Disease using Structural Magnetic Resonance Imaging. AMIA Annual Symposium proceedings AMIA Symposium. 2010;2010:542–6. Epub 2011/02/25. 21347037 PMC3041374

[33] Kloppel S, Stonnington CM, Chu C, Draganski B, Scahill RI, Rohrer JD, et al Automatic classification of MR scans in Alzheimer's disease. Brain: a journal of neurology. 2008;131(Pt 3):681–9. Epub 2008/01/19. 18202106 10.1093/brain/awm319 PMC2579744

[34] Aguilar C, Westman E, Muehlboeck JS, Mecocci P, Vellas B, Tsolaki M, et al Different multivariate techniques for automated classification of MRI data in Alzheimer's disease and mild cognitive impairment. Psychiatry research. 2013;212(2):89–98. Epub 2013/04/02. 10.1016/j.pscychresns.2012.11.005 23541334

[35] Beheshti I, Demirel H. Feature-ranking-based Alzheimer's disease classification from structural MRI. Magnetic resonance imaging. 2016;34(3):252–63. Epub 2015/12/15. 10.1016/j.mri.2015.11.009 26657976

[36] Dale A, Fischl B, Sereno M. Cortical surface-based analysis i: Segmentation and surface reconstruction. NeuroImage. 1999;9(2):179–94. 10.1006/nimg.1998.0395 9931268

[37] Dale A, Sereno M. Improved localization of cortical activity by combining EEG and MEG with MRI cortical surface reconstruction: a linear approach. J Cogn Neurosci 1993;5:162–76. 10.1162/jocn.1993.5.2.162 23972151

[38] Fischl B, Sereno M, Dale A. Cortical surface-based analysis-ii: inflation, flatting, and a surface-based coordinate system. NeuroImage. 1999;9:195–207. 10.1006/nimg.1998.0396 9931269

[39] Desikan R, Segonne F, Fischl B, Quinn B, Dickerson B, Blacker D, et al An automated labeling system for subdividing the human cerebral cortex on MRI scans into gyral based regions of interest. NeuroImage. 2006;31(3):968–80. 10.1016/j.neuroimage.2006.01.021 16530430

[40] Fischl B, van der Kouwe A, Destrieux C, Halgren E, Segonne F, Salat D, et al Automatically Parcellating the Human Cerebral Cortex. Cerebral cortex (New York, NY: 1991). 2004;14:11–22. 10.1093/cercor/bhg087 14654453

[41] Vercauteren T, Pennec X, Perchant A, Ayache N. Symmetric log-domain diffeomorphic Registration: a demons-based approach. Medical image computing and computer-assisted intervention: MICCAI International Conference on Medical Image Computing and Computer-Assisted Intervention. 2008;11(Pt 1):754–61. Epub 2008/11/05. 10.1007/978-3-540-85988-8_90 18979814

[42] Fletcher PT, Lu C, Joshi S, editors. Statistics of shape via principal geodesic analysis on Lie groups. IEEE Computer Society Conference on Computer Vision and Pattern Recognition, vol 1, page I-95–I-101; 2003.

[43] Bengio Y, Paiement J, Vincent P, Delalleau O, Roux N, Ouimet M. Out-of-Sample Extensions for LLE, ISPMAP, MDS, Eigenmaps, and Spectral Clustering Advances in Neural Information Processing Systems. Cambridge, MA, USA: The MIT Press; 2004.

[44] Casanova R, Whitlow CT, Wagner B, Williamson J, Shumaker SA, Maldjian JA, et al High dimensional classification of structural MRI Alzheimer’s disease data based on large scale regularization. Frontiers in Neuroinformatics. 2011;5:Article 22. 10.3389/fninf.2011.00022 PMC3193072 22016732

[45] Westman E, Muehlboeck JS, Simmons A. Combining MRI and CSF measures for classification of Alzheimer's disease and prediction of mild cognitive impairment conversion. NeuroImage. 2012;62(1):229–38. Epub 2012/05/15. 10.1016/j.neuroimage.2012.04.056 22580170

[46] Willette AA, Calhoun VD, Egan JM, Kapogiannis D. Prognostic classification of mild cognitive impairment and Alzheimer's disease: MRI independent component analysis. Psychiatry research. 2014;224(2):81–8. Epub 2014/09/10. 10.1016/j.pscychresns.2014.08.005 25194437 PMC4586157

[47] Zhou Q, Goryawala M, Cabrerizo M, Wang J, Barker W, Loewenstein DA, et al An Optimal Decisional Space for the Classification of Alzheimer's Disease and Mild Cognitive Impairment. IEEE Transactions on Biomedical Engineering. 2014;61(8):2245–53. 10.1109/TBME.2014.2310709 25051543

[48] Fan Y, Batmanghelich N, Clark CM, Davatzikos C. Spatial patterns of brain atrophy in MCI patients, identified via high-dimensional pattern classification, predict subsequent cognitive decline. NeuroImage. 2008;39(4):1731–43. Epub 2007/12/07. 10.1016/j.neuroimage.2007.10.031 18053747 PMC2861339

[49] Querbes O, Aubry F, Pariente J, Lotterie JA, Demonet JF, Duret V, et al Early diagnosis of Alzheimer's disease using cortical thickness: impact of cognitive reserve. Brain: a journal of neurology. 2009;132(Pt 8):2036–47. Epub 2009/05/15. 19439419 10.1093/brain/awp105 PMC2714060

[50] Lillemark L, Sorensen L, Pai A, Dam EB, Nielsen M. Brain region's relative proximity as marker for Alzheimer's disease based on structural MRI. BMC medical imaging. 2014;14:21 Epub 2014/06/04. 10.1186/1471-2342-14-21 24889999 PMC4048460

[51] Magnin B, Mesrob L, Kinkingnehun S, Pelegrini-Issac M, Colliot O, Sarazin M, et al Support vector machine-based classification of Alzheimer's disease from whole-brain anatomical MRI. Neuroradiology. 2009;51(2):73–83. Epub 2008/10/11. 10.1007/s00234-008-0463-x 18846369

[52] Tenenbaum JB, de Silva V, Langford JC. A global geometric framework for nonlinear dimensionality reduction. Science (New York, NY). 2000;290(5500):2319–23. Epub 2000/12/23. 10.1126/science.290.5500.2319 11125149

[53] Davatzikos C, Bhatt P, Shaw LM, Batmanghelich KN, Trojanowski JQ. Prediction of MCI to AD conversion, via MRI, CSF biomarkers, and pattern classification. Neurobiology of aging. 2011;32(12):2322 e19–27. Epub 2010/07/03. 10.1016/j.neurobiolaging.2010.05.023 PMC2951483 20594615

[54] Zhang D, Shen D, the AsDNI. Predicting Future Clinical Changes of MCI Patients Using Longitudinal and Multimodal Biomarkers. PLoS One. 2012;7(3):e33182 10.1371/journal.pone.0033182 22457741 PMC3310854

[55] Shaffer JL, Petrella JR, Sheldon FC, Choudhury KR, Calhoun VD, Coleman RE, et al Predicting cognitive decline in subjects at risk for Alzheimer disease by using combined cerebrospinal fluid, MR imaging, and PET biomarkers. Radiology. 2013;266(2):583–91. Epub 2012/12/13. 10.1148/radiol.12120010 23232293 PMC3558874

[56] C R, H FC, S KM, R SR, W JD, R SM, et al Alzheimer's disease risk assessment using large-scale machine learning methods. PLoS One. 2013;8(11):e77949 10.1371/journal.pone.0077949 24250789 PMC3826736

[57] Moradi E, Pepe A, Gaser C, Huttunen H, Tohka J. Machine learning framework for early MRI-based Alzheimer's conversion prediction in MCI subjects. NeuroImage. 2015;104:398–412. Epub 2014/10/15. 10.1016/j.neuroimage.2014.10.002 25312773 PMC5957071

[58] Misra C, Fan Y, Davatzikos C. Baseline and longitudinal patterns of brain atrophy in MCI patients, and their use in prediction of short-term conversion to AD: results from ADNI. NeuroImage. 2009;44(4):1415–22. Epub 2008/11/26. 10.1016/j.neuroimage.2008.10.031 19027862 PMC2648825

[59] Shaw LM, Vanderstichele H, Knapik-Czajka M, Clark CM, Aisen PS, Petersen RC, et al Cerebrospinal fluid biomarker signature in Alzheimer's disease neuroimaging initiative subjects. Annals of neurology. 2009;65(4):403–13. Epub 2009/03/20. 10.1002/ana.21610 19296504 PMC2696350

[60] Musiek ES, Chen Y, Korczykowski M, Saboury B, Martinez PM, Reddin JS, et al Direct comparison of fluorodeoxyglucose positron emission tomography and arterial spin labeling magnetic resonance imaging in Alzheimer's disease. Alzheimer's & dementia: the journal of the Alzheimer's Association. 2012;8(1):51–9. Epub 2011/10/25. 10.1016/j.jalz.2011.06.003 PMC3264701 22018493

[61] Fox NC, Scahill RI, Crum WR, Rossor MN. Correlation between rates of brain atrophy and cognitive decline in AD. Neurology. 1999;52(8):1687–9. Epub 1999/05/20. 10331700 10.1212/wnl.52.8.1687

[62] Buckner RL, Snyder AZ, Shannon BJ, LaRossa G, Sachs R, Fotenos AF, et al Molecular, structural, and functional characterization of Alzheimer's disease: evidence for a relationship between default activity, amyloid, and memory. The Journal of neuroscience: the official journal of the Society for Neuroscience. 2005;25(34):7709–17. Epub 2005/08/27. 16120771 10.1523/JNEUROSCI.2177-05.2005 PMC6725245

[63] Petrella JR, Coleman RE, Doraiswamy PM. Neuroimaging and early diagnosis of Alzheimer disease: a look to the future. Radiology. 2003;226(2):315–36. Epub 2003/02/04. 10.1148/radiol.2262011600 12563122

[64] Poulin SP, Dautoff R, Morris JC, Barrett LF, Dickerson BC. Amygdala atrophy is prominent in early Alzheimer's disease and relates to symptom severity. Psychiatry research. 2011;194(1):7–13. Epub 2011/09/17. 10.1016/j.pscychresns.2011.06.014 21920712 PMC3185127

[65] Cho H, Kim JH, Kim C, Ye BS, Kim HJ, Yoon CW, et al Shape changes of the basal ganglia and thalamus in Alzheimer's disease: a three-year longitudinal study. Journal of Alzheimer's disease: JAD. 2014;40(2):285–95. Epub 2014/01/15. 10.3233/JAD-132072 24413620

[66] Scahill RI, Schott JM, Stevens JM, Rossor MN, Fox NC. Mapping the evolution of regional atrophy in Alzheimer's disease: unbiased analysis of fluid-registered serial MRI. Proceedings of the National Academy of Sciences of the United States of America. 2002;99(7):4703–7. Epub 2002/04/04. 10.1073/pnas.052587399 11930016 PMC123711

[67] Eskildsenemail S, Gyldensted L, Nagenthiraja K, Frandsen J, Rodell A, Gyldensted C, et al Increased oxygen extraction capacity in the basal ganglia and thalamus of people with Alzheimer's disease. Alzheimer's and Dementia. 2013;9(4):Suppl pp 102.

